# First Insights into the Anti-Inflammatory Potential of *Colliguaja odorifera* Molina Leaf Extracts and Their Isolated Phenolic Compounds

**DOI:** 10.3390/plants14243839

**Published:** 2025-12-17

**Authors:** Amy Figueroa, Ana Mutis, Emilio Hormazabal, Olga Rubilar, Edward Hermosilla, João Henrique Ghilardi Lago, Andrés Quiroz, Javier Espinoza

**Affiliations:** 1 Doctorado en Ciencias de Recursos Naturales, Universidad de La Frontera, Temuco 4811230, Chile; a.figueroa07@ufromail.cl; 2 Laboratorio de Ecología Química, Departamento de Ciencias Químicas y Recursos Naturales, Universidad de La Frontera, Casilla 54-D, Temuco 4811230, Chile; ana.mutis@ufrontera.cl (A.M.); javier.espinoza@ufrontera.cl (J.E.); 3 Centro de Excelencia en Investigación Biotecnológica Aplicada al Medio Ambiente (CIBAMA), Universidad de La Frontera, Casilla 54-D, Temuco 4811230, Chile; 4 Departamento de Ciencias Químicas y Recursos Naturales, Universidad de La Frontera, Casilla 54-D, Temuco 4811230, Chile; 5 Centro de Ciências Naturais e Humanas, Universidade Federal do ABC, Santo André 09280-560, Brazil; joao.lago@ufabc.edu.br

**Keywords:** *Colliguaja odorifera*, Euphorbiaceae, phenolic compounds, lipoxygenase inhibition

## Abstract

Despite the ethnobotanical significance of Chilean *Colliguaja* species, research on their biological activities and phytochemical composition remains limited. Among these species, *Colliguaja odorifera* Molina (Euphorbiaceae), traditionally used in folk medicine to alleviate toothaches, stands out for its potential for medicinal applications. This study aims to investigate the anti-inflammatory activity of the *C. odorifera* leaf extracts and their secondary metabolites isolated from the most active extract. A hydroalcoholic extract of *C. odorifera* leaves was prepared, and subsequently ethyl acetate (EA-E), *n*-butanol (B-E), and water (W-E) extracts were obtained by liquid–liquid partition. The extracts were first evaluated for their ability to inhibit lipoxygenase, and the most active extract was subsequently tested for hyaluronidase (HA) and secretory phospholipase A2 (sPLA_2_). The most active extract was EA-E, with IC_50_ values of 11.75, 31.09, and 6.60 µg/mL for anti-LOX activity, hyaluronidase, and sPLA_2_, respectively. This extract was analyzed by chromatography coupled to mass spectrometry and ^1^H and ^13^C NMR spectroscopy, allowing the identification, for the first time, of shikimic acid, gallic acid, methyl gallate, ethyl gallate, and a putative galloyl-luteolin. These results suggest that *C. odorifera* is a promising candidate for the development of natural alternatives to nonsteroidal anti-inflammatory drugs.

## 1. Introduction

The Euphorbiaceae family, one of the largest families of flowering plants, is represented by approximately 7500 species distributed across 300 genera and 50 tribes [1]. In Chile, this family is represented by 45 taxa, including 35 endemic and native species distributed across seven genera: *Adenopeltis*, *Argythamnia*, *Avellanita*, *Colliguaja*, *Croton*, *Chiropetalum*, *Dysopsis*, and *Euphorbia* [2,3]. Since ancient times, the Euphorbiaceae family has been known for its diverse medicinal properties. Over 214 species with medicinal properties have been identified in Chile, and there is a rich history of using some Chilean species from the Euphorbiaceae family in traditional medicine, making them a vital part of Chilean cultural heritage [2,3]. Notwithstanding the above, the species of *Colliguaja*, such as *Colliguaja dombeyana* A. Juss., *Colliguaja integerrima* Gillies & Hook, *Colliguaja salicifolia* Gillies & Hook, and *Colliguaja odorifera* Molina, which are cataloged as endemic [4], have not been as extensively researched or documented in this regard. The Mapuche ethnic group used to call these *Colliguaja* species “Collihuayu,”. *C. odorifera* is a woody monoecious shrub distributed from 23°30′ to 35°32′ S, covering different ecological environments. The staminate (male) flowers are arranged in aments, with pistillate (female) flowers located at their base. This species is notable for the fragrant aroma emitted from its wood. Its fruit consists of a self-dehiscent, dry capsule that explodes in summer when mature, dispersing seeds a few meters away from the parent plant. The species possesses long-lasting renewal buds in a subterranean structure known as a lignotuber. This feature suggests that *C. odorifera* possesses a rich evolutionary history, with an ancient population that supports a diverse age structure, indicating its resilience and adaptability to changing environments [5]. Murillo [6] and Espinoza [7] noted that *C. odorifera* has a milky juice used to induce decayed teeth to fall out and relieve toothaches. This white latex comes off when the plant suffers cuts in various parts. Later, Gusinde [8] and de Mössbach [9] documented that latex from cut roots was used to poison arrows and spears. Moreover, Vitorino et al. [10] state that the main parts of the *C. integerrima* plant used in traditional medicine are the aerial organs and the white latex, both of which have been used as plasters for analgesic purposes [11]. A decoction of the plant was also utilized as a disinfectant for vaginal infections [9]. Bittner et al. [12] documented that *C. odorifera* was used as an analgesic. Cordero et al. [13] reported that the four *Colliguaja* species are used for livestock feed. The literature does not specify which part of the plant was used for toothaches or vaginal infections. However, Bittner et al. [12] reported the antimicrobial activity of leaves and stems of *C. salicifolia*, *C. odorifera*, and *C. integerrima* against *Escherichia coli*, *Staphylococcus aureus*, and *Staphylococcus lutea*, and leaves and stems of the last two species showed anti-tumor activity over lymphocytic leukemia in mice, and human nasopharyngeal carcinoma. Alvarez et al. [14] reported that an aqueous infusion of the aerial parts of *C. integerrina* showed a moderate diuretic activity.

Chemical studies on the *Colliguaja* genus have been limited to the identification of secondary metabolites, which are indicative of phylogenetic relationships. However, Vitorino et al. [10] dedicate a chapter to reviewing the principal chemical constituents and bioactive compounds of *C. integerrima*, highlighting the presence of flavonoids such as kaempferol, daidzein, pelargonidin, and delphinidin. On the contrary, *C. odorifera* has received little attention regarding its phytochemistry. In this respect, cis-1,4-polyisoprene, *n*-heptacosane (17%), and *n*-nonacosane (82%) were identified in stems and leaves of *C. odorifera* when they were extracted with dichloromethane and acetone [15,16]. Later, Bittner et al. [3] identified known triterpenes such as lupeol, ursolic acid, *β*-sitosterol, and oleanolic acid, and also flavonoid glycosides as quercetin-3-O-glucoside, quercetin-3-O-glucosyl-rhamnoside, and quercetin-3-*O*-rhamnosyl-arabinoside. The compounds were extracted from the whole plant through mixture of methanol/dichloromethane/hexane (1:1:1). Regarding to the bioactivity of these compounds, lupeol has been extensively studied for its inhibitory effects on inflammation under in vitro and in animal models of inflammation [17], and quercetin-3-*O*-glucoside has shown an anti-lipoxygenase effect [18]. However, there are not reports focused in the chemical basis related to the medicinal uses of *C. odorifera*.

Consequently, this study aimed to evaluate the anti-inflammatory activities of *C. odorifera* leaf extracts using in vitro assays and to characterize the secondary metabolites of the most active extract.

## 2. Results

### 2.1. LOX Inhibitory Activity of Colliguaja Odorifera Leaf Extracts

Compounds that inhibit the LOX enzyme are potential candidates for anti-inflammatory activity. Ethyl acetate extract and butanol extract (B-E) derived from the hydroalcoholic extract (HA-E) of *C. odorifera* exhibited differing levels of LOX inhibitory activity. EA-E demonstrated the highest LOX inhibition among the extracts, with an IC_50_ value of 11.75 ± 1.86 µg/mL. This was followed by the B-E, which exhibited an IC_50_ of 42.50 ± 10.61 µg/mL. The crude extract (HA-E) showed an IC_50_ of 64.56 ± 12.92 µg/mL, and W-E did not elicit an anti-LOX activity (Table 1).

The phenolic compounds, shikimic acid and gallic acid, isolated from EA-E, exhibited significant LOX inhibitory activity, with IC_50_ values of 0.97 ± 0.04 µg/mL and 0.37 ± 0.15 µg/mL, respectively. The positive controls, quercetin and naproxen, elicited 50% inhibition of LOX activity at concentrations of 0.48 ± 0.03 µg/mL (1.59 µM) and 0.56 ± 0.02 µg/mL (2.5 µM), respectively.

The EA-E of *C. odorifera* was selected for further analysis due to its better performance in the anti-LOX assay.

### 2.2. sPLA_2_ and Hyaluronidase Inhibitory Activities of C. odorifera Leaf Extracts

To assess the anti-inflammatory effects of *C. odorifera* leaf extracts and their compounds, the inhibitory activities against the enzymes sPLA_2_ and hyaluronidase, which depolymerize hyaluronic acid (HA), can also be evaluated. Gallic acid showed the best performance in inhibiting sPLA_2_-V activity (IC_50_ 11.16 μg/mL). It was statistically different from quercetin and EA-E, with IC_50_ values of 23.48 μg/mL and 31.09 μg/mL, respectively. Moreover, gallic acid exhibited the highest hyaluronidase activity (IC_50_ 1.76 µg/mL), which was statistically similar to that of the positive control (quercetin, IC_50_ 1.80 µg/mL). Ethyl acetate extract showed the lowest hyaluronidase inhibition (IC_50_, 6.6 µg/mL), and shikimic acid did not elicit anti-sPLA_2_-V and anti-hyaluronidase activities (Table 2).

### 2.3. Chemical Characterization of the Ethyl Acetate Extract (EA-E)

**Figure 1:**
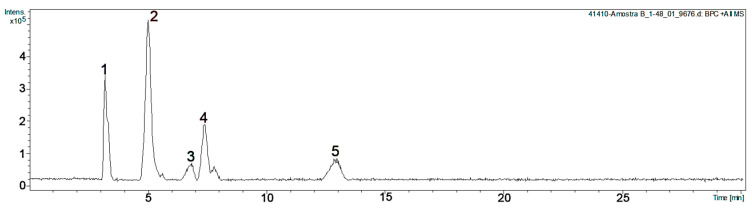
HPLC-MSMS chromatogram of the ethyl acetate extracts from *Colliguaja odorifera* leaves. 1: Shikimic acid; 2: gallic acid; 3: methyl gallate; 4: ethyl gallate; 5: galloyl luteolin.

From 898 g of dry leaves of *C. odorifera*, 236 g of ethyl acetate extract (EA-E), 218 g of butanol extract (B-E), and 95 g of water residue (W-E) were obtained, yielding 26.3% 24.3%, and 10.6%, respectively. The EA-E of *C. odorifera* exhibited the strongest LOX, hyaluronidase, and sPLA_2_ inhibition, according to the results presented in Table 1 and Table 2. Figure 1 and Table 3 show the chromatogram and the identification of the compound present in EA-E from *C. odorifera* leaves. The main compounds were tentatively identified by co-chromatography with authentic standards, by comparing their UV spectra, mass spectrometric data, and NMR data, and by consulting the PubChem database and various scientific literature sources. Positive ionization data were used for identification, and sodium was used to improve detection sensitivity and accuracy.

Peak 1 showed a [M + Na]^+^ ion at *m*/*z* 197 corresponding to *m*/*z* 174 in addition to Na^+^ (*m*/*z* 23), and a fragment ion at *m*/*z* 157, possibly due to the loss of a hydroxyl group (-OH). This compound was isolated by column chromatography from EA-E, and NMR data allowed it to be assigned to this peak as shikimic acid.

Peak 2 showed an [M + Na]^+^ ion at *m*/*z* 193 corresponding to *m*/*z* 170 in addition to Na^+^, and fragment ions at *m*/*z* 171 and 153 would correspond to the protonation of the neutral molecule and the loss of the -OH group, respectively. NMR data of this isolated compound were consistent with the presence of gallic acid.

Peak 3 showed an [M + H]^+^ ion at *m*/*z* 185, and the NMR data of this isolated compound were consistent with those of methyl gallate.

Peak 4 showed an [M + Na]^+^ ion at *m*/*z* 221 corresponding to the molecular ion *m*/*z* 198 in addition to Na^+^. The fragment ion at *m*/*z* 199 would correspond to the protonation of the neutral molecule. NMR data of this isolated compound were consistent with the presence of ethyl gallate.

**Figure 2:**
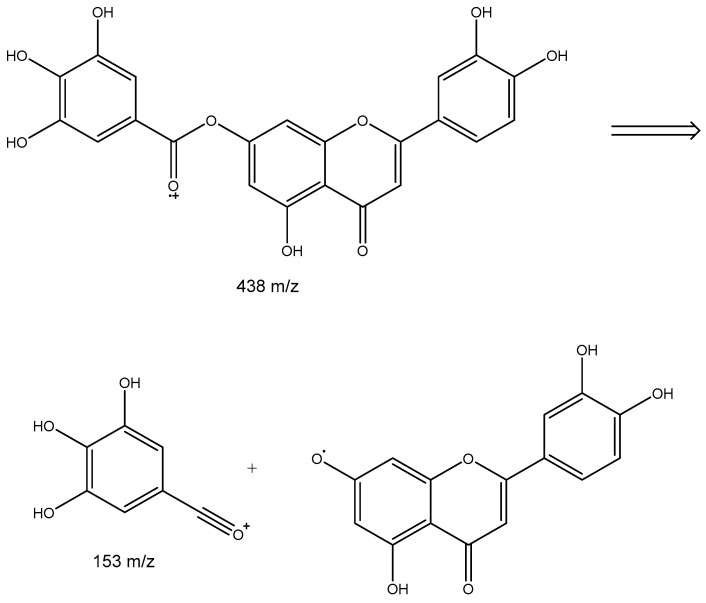
Proposed fragmentation of galloyl luteolin.

Peak 5 would correspond to a putative galloy luteolin. Figure 2 shows the possible fragmentation of this compound, showing a [luteolin + Na]^+^ ion at *m*/*z* 309 and a [gallic acid-OH]^+^ ion at 153.

## 3. Discussion

LOX is a key enzyme that regulates the conversion of arachidonic acid into leukotrienes, which mediate inflammatory responses. Inhibiting LOX activity reduces leukotriene production (pro-inflammatory mediators), exerting an anti-inflammatory effect [19]. LOXs are categorized with respect to their positional specificity of arachidonic acid oxygenation. In our case, the soybean Type I-B LOX A oxygenates linoleic acid at C-15 [20]. Loncaric et al. [21] reviewed the inhibitory LOX activity of plant extracts from various families, highlighting their effectiveness in inhibiting soybean lipoxygenases. They reported IC_50_ values ranging from 0.01 µg/mL to 899.97 µg/mL for the different extracts and plant parts. This study represents the first report on the anti-inflammatory activity of the Chilean Euphorbiaceae *C. odorifera.* The ethyl acetate extract of *C. odorifera* leaves elicited a strong anti-LOX response (IC_50_ = 11.75 µg/mL).

According to Table 4, the anti-LOX IC_50_ ethyl acetate extract of C. odorifera leaves is better than those elicited by other Euphorbiaceae species. The comparisons are challenging due to the use of different extraction solvents and natural compounds, such as baicalein and nordihydroguaiaretic acid, used as positive controls. On the contrary, the anti-LOX activity elicited here by EA-E was compared with that of naproxen, a nonsteroidal anti-inflammatory drug (NSAID) used to relieve pain, reduce inflammation, and act as an antipyretic. Like other non-selective NSAIDs, naproxen exerts its clinical effects by inhibiting cyclooxygenase-1 and cyclooxygenase-2, thereby decreasing prostaglandin synthesis. However, reports have shown that naproxen can inhibit the LOX enzyme [22].

Moreover, our result (anti-LOX IC_50_ = 11.75 µg/mL) is even better than those obtained for different families (Apiaceae and Lamiaceae), but less active than species from the families Clisiaceae and Menispermaceae (Table 5).

All these investigations attributed the anti-LOX activity to total flavonoid content, and the solvent most commonly used facilitated the extraction of flavonoids and phenolic compounds [33,34]. Numerous studies have provided robust evidence for the potent anti-inflammatory effects of ethyl acetate fractions and the compounds they contain [21,35,36]. In the present study, two phenolic compounds, shikimic acid and gallic acid, identified in EA-E, exhibited strong anti-LOX activity, with concentrations of 5.6 µM and 2.2 µM, respectively, comparable to those of the positive controls, quercetin (1.6 µM) and Naproxen (2.5 µM).

Another approach to assessing the anti-inflammatory effects of compounds is to evaluate their inhibitory activity against the enzyme sPLA_2_. The PLA_2_ superfamily has been classified into 16 groups (groups I to XVI) [37]. PLA2 groups can also be divided into six subfamilies namely into secreted PLA2s (sPLA_2_s) (groups I, II, III, V, IX, X, XI, XII, XIII and XIV), cytosolic PLA2s (cPLA2s) (group IV), Ca^2+^-independent PLA2s (iPLA2s) (group VI), platelet-activating factor acetylhydrolase PLA2s (PAF-AH PLA2s) (groups VII and VIII), lysosomal PLA2s (LPLA2s) (group XV) and adipose-tissue-specific PLA2s (AdPLA2s) (group XVI) [38,39]. This enzyme plays a critical role in the inflammatory response by releasing fatty acids, such as arachidonic acid, from cellular membrane phospholipids. Arachidonic acid is a key precursor in the synthesis of pro-inflammatory mediators, including prostaglandins and leukotrienes [40]. Furthermore, compounds that inhibit sPLA_2_ are considered promising candidates for the development of new anti-inflammatory drugs. Group V of sPLA_2_ was selected here because: (a) it is a more versatile isoform and plays a central role in amplifying the inflammatory response, (b) This enzyme binds to cell membranes via interfacial binding and to proteoglycans, and (c) prefers to hydrolyze lipids with unsaturated fatty acids in the sn-2 position having a low degree of unsaturation, such as oleic acid (C18:1) and linoleic acid (C18:2) [37]. The role of sPLA_2_-V as an enzyme initiating eicosanoid generation is well documented [41]. However, to our knowledge, no work has been conducted on Euphorbiaceae species to measure the inhibitory activity of this enzyme. The ability to inhibit sPLA_2_ activity of EA-E from *C. odorifera* (IC_50_ of 31.09 µg/mL) was comparable to the inhibition shown by *Ribes nigrum* (27.7 μg/mL) [42], *Ononis spinose* (39.44 μg/mL) [39], and *Boerhaavia diffusa* (17.8 μg/mL) [43]. These results show that EA-E from *C. odorifera* leaves falls within the range of hyaluronidase activity reported for different plant species.

Hyaluronidase depolymerizes hyaluronic acid (HA), thereby regulating the size and concentration of HA chains and influencing various pathological processes. They are crucial targets for the development of new agents to inhibit inflammation [44]. As far as we know, there are no reports of anti-HA activity of Euphorbiaceae species. In this study, EA-E from *C. odorifera* leaves showed HA inhibition with an IC_50_ of 6.6 µg/mL. Gallic acid and quercetin showed the highest inhibition, with IC_50_ values of 1.76 and 1.31 µg/mL, very similar to the positive control, quercetin (1.80 and 0.17 µg/mL) (Table 2). However, HA inhibition by plant extracts has been reported in other families. Paun et al. [27] reported an IC_50_ of 24.6 ± 1.5 µg/mL for HA elicited from a polyphenolic fraction of the aerial parts of *E. planum* L. (Apiaceae). Additionally, Studzińska-Sroka et al. [45] examined the anti-inflammatory activity of a hydroalcoholic extract from Galinsoga parviflora (Asteraceae), demonstrating anti-hyaluronidase activity with an IC_50_ of 0.47 mg/mL, which is stronger than that of the positive control, kaempferol, with an IC_50_ of 0.78 mg/mL.

In the present study, shikimic acid was not active against sPLA_2_ V and HA. Balsinde et al. [46] reported that an indole derivative and a naphthalenic derivative, both aromatic, inhibited phospholipase A2 activity. This suggests that the absence of an aromatic ring in shikimic acid may explain its lack of activity. A similar explanation can be applied to the lack of effect of shikimic acid on HA. The main anti-inflammatory drugs, such as celecoxib, nimesulide, indomethacin, and fenoprofen, among others, reported as HA inhibitors [47], possess one or more aromatic rings. This suggests that the planarity of a specific part of the molecule may be essential for the activity of certain compounds on HA.

The promising anti-inflammatory activity exhibited by *C. odorifera* leaves can be attributed to the phenolic compounds identified in the ethyl acetate extract. Among the chemical compounds capable of inhibiting LOX catalytic activity, the actions of different classes of phenolic compounds, such as flavonoids, catechins, carotenoids, and isoflavones, have been studied [48,49].

In this respect, the phenolic acids, shikimic and gallic acid, and phenol esters, methyl gallate and ethyl gallate, were identified in the EA-E from *C. odorifera* leaves in the present study. Shikimic acid possesses noteworthy biological properties, exhibiting antioxidant, antibacterial, anti-inflammatory, and analgesic activities [50]. Our results showed that shikimic acid exhibited LOX inhibitory activity similar to Naproxen (IC_50_, 0.56 ± 0.02 µg/mL), consistent with the literature. Gallic acid is widely utilized in the pharmaceutical sector. It is effective in inhibiting cardiovascular diseases, Alzheimer’s disease, and Parkinson’s disease [51] and exhibits anti-inflammatory activity [52]. Methyl gallate is commonly found in the Euphorbiaceae family. Research has demonstrated that methyl gallate exhibits various pharmacological activities, including anti-tumor, anti-inflammatory, and anti-microbial effects [53]. Recently, anti-inflammatory and anti-nociceptive activities have been associated with a high abundance of methyl gallate, 1,2,3,4,6-pentagalloylhexose, and quercitrin [54]. Ethyl gallate (EG) has been shown to exhibit anti-inflammatory activity in cell organelles, which may be beneficial in the treatment of inflammatory diseases. In India, *Pistacia integerrima* Linn. galls, which naturally contain EG, are used in traditional medicine [55,56].

**Figure 3:**
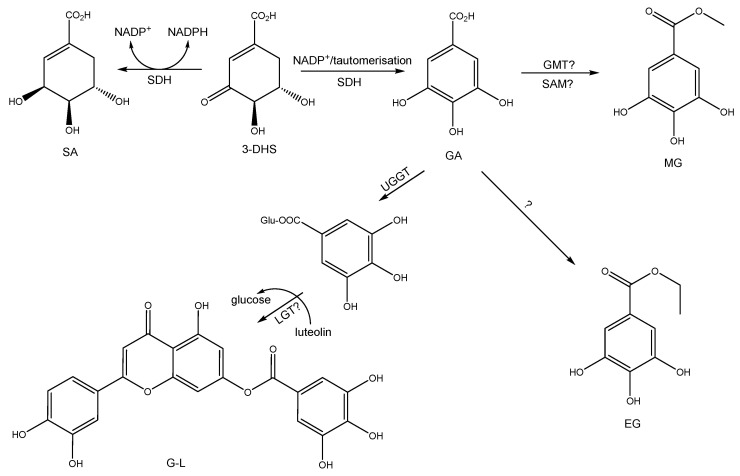
Biosynthetic pathway proposed for gallic acid (GA), shikimic acid (SA), methyl gallate (MG), ethyl gallate (EG), and galloyl-luteolin (GL). SDH, SA dehydrogenase; GMT, galloyl-O-methyltransferase; UGGT, UDP-glucose: gallate 1-O-galloyltransferase; LGT, luteolin-gallloyltransferase in *Colliguaja odorifera*; SAM: S-adenosyl methionine; 3-DHS: 3-dehydro shikimic acid; ? is a suggestion, because it has not determined or isolated the proper enzyme or coenzyme.

Based on the report by Huang et al. [57], we propose a hypothetical biosynthetic mechanism, as shown in Figure 3. Shikimic acid can be formed from 3-dehydroshikimate and NADPH, catalyzed by SA dehydrogenase (SDH) [58,59]. Gallic acid is mainly derived from the dehydrogenation of 3-DHS by the action of SDH [60]. Ossipov et al. [61] have suggested that NADP+ participates as a cofactor in this reaction, and a tautomerization of the keto-enolic type is proposed to form gallic acid. The formation of galloyl-luteolin could be similar to that proposed by Huang et al. [57] in *Camellia sinensis*, where, in the first step, gallic acid is glucosylated through UGGT. Then, a putative luteolin-galloyl-transferase would form galloyl-luteolin, similar to how gallic acid glucosylated and catechin are synthesized into gallic acid glucosylated and catechin [57].

GA appears to be a precursor of most of the compounds identified in the EA-E, and is an essential precursor for the putative galloyl-luteolin biosynthesis. This fact could be related to the vast amount of this compound found in the EA-E. GA was the most abundant compound in EA-E, with an estimated concentration of 85.0 mg/g, as determined by a calibration curve using the respective pure standard. This finding may be related to the formation of the three gallate derivatives, including methyl gallate, ethyl gallate, and galloyl-luteolin. This investigation constitutes the first report of the anti-inflammatory potential of *C. odorifera*. However, the butanol extract also showed strong anti-LOX activity; further work is ongoing to identify the responsible compounds.

## 4. Materials and Methods

### 4.1. Plant Collection and Extracts Obtention

Plants of *C. odorifera* were collected between the Valparaíso Region and the La Araucanía Region (Chile) in October 2020. The plant materials were then carefully transferred to the Chemical Ecology Laboratory at the Universidad de La Frontera in Temuco, Chile. The species was identified as *Colliguaja odorifera* Molina by the Botany Department at Universidad de Concepción, and voucher specimens were deposited in the Universidad de Concepción’s herbarium (Herbarium code No. CONC 13817).

Leaves (898 g) of *C. odorifera* were thoroughly washed four times with fresh tap water and once with distilled water to remove dirt, soil, and contaminants. They were air-dried at room temperature (25 ± 2 °C) for 14 days, then dried for an additional 24 h at 30 ± 2 °C in a laboratory dryer before being ground into a coarse powder. The leaves were defatted with CHCl_3_, then the defatted leaves were macerated with ethanol (80%) in distilled water [21,61,62] for seven days with periodic stirring. After maceration, the mixture was filtered, and the solution was concentrated in a rotary evaporator at reduced pressure until the ethanol was eliminated. The remaining water was removed by freeze-drying to obtain the hydro-alcoholic extract (HA-E). This extract was subsequently dissolved in 1 L of distilled water and partitioned between ethyl acetate, *n*-butanol, and water. Each partitioning step was performed three times in a 1:1 ratio of sample to solvent [63,64,65]. After each organic phase was dried with anhydrous Na_2_SO_4_, vacuum-filtered, and evaporated to dryness using a rotary evaporator, three extracts were obtained: Ethyl acetate extract (EA-E), *n*-butanol extract (B-E), and water extract (W-E). These extracts were subjected to enzymatic bioassays and phytochemical characterization. All solvents used in this study were purchased from Sigma-Aldrich (St. Louis, MO, USA) and were of chromatographic grade, ensuring their purity.

### 4.2. Inhibitory LOX Activity

The inhibitory LOX assay was conducted according to the procedure established by Lyckander and Malterud [66]. The lipoxygenase Type I-B LOX A from *Glycine max* L. (soybean) (CAS: 9029-60-1) (15-LOX) with an optimal pH of 8.0–9.0 and linoleic acid used here, was obtained from Sigma-Aldrich (Steinheim, Germany).

A linoleic acid ethanol solution (268 µM) was prepared, and 50 µL of this solution was used to prepare concentrations ranging from 0.6 to 26 µg/mL for the HA-E sample, and from 0.6 to 12.9 µg/mL for the EA-E and B-E samples. Then the volume for each concentration was adjusted to 1 mL with 200 mM borate buffer (pH 8.75) containing 0.005% Tween 80. The reaction was initiated by adding 1.5 µL of Soybean 1-LOX (948 U), equivalent to 0.054 g/mL. Enzymatic activity was monitored by measuring the increase in absorbance at 234 nm for 4 min at 37 °C. Each enzyme measurement for each dilution was repeated three times. All tests were performed in triplicate. For this purpose, three samples were taken from each extract. For each of them, enzymatic activity was determined as described above. Naproxen was used as the positive control. The percentage inhibition of enzyme activity was calculated by comparing the absorbance of the samples to that of the blank, using Equation (1) [67], where A_0_ is the absorbance of the blank, and A_1_ is the absorbance of the sample.

% inhibition = [(A_0_ − A_1_)/A_0_] × 100 (1)

The percentage of inhibition was plotted against sample concentration to calculate the IC_50_ value.

### 4.3. Screening Assay for Phospholipase A_2_ Inhibitors (Type V)

The phospholipase A_2_ inhibitory activity was determined using the phospholipase A_2_ (sPLA_2_) inhibitor detection assay kit (Type V). This kit was purchased from Cayman Chemical Co. (Ann Arbor, MI, USA). Briefly, 10 μL of *C. odorifera* extracts (HA-E, EA-E, B-E, and W-E, separately) were pre-incubated with 25 mM Tris-HCl buffer at pH 7.5, including 10 μL of phospholipase A_2_. The reaction was initiated by adding 200 μL of a 1.66 mM diheptanoyl thio-PC substrate solution. The plate was then shaken for 30 s and incubated at 25 °C for 15 min. Then, 10 µL of 5,5′-dithiobis(2-nitrobenzoic acid) (DTNB) was added to stop the enzymatic reaction and allow color development. The reaction mixture was stirred on a shaker for one minute, and the absorbance was subsequently measured at 405 nm with a spectrophotometer (METASH, UV-1500B; Shanghai Metash Instrument Co., Shanghai, China). According to the kit instructions, the sPLA2 activity was evaluated with extract concentrations ranging from 27 to 108 μg/mL. Arachidonic thioester phosphatidylcholine was used as the positive control [68]. Moreover, quercetin was also used as a positive control because it is known for its anti-inflammatory activity through modulation of enzymes in the arachidonic acid cascade [69]. All tests were performed in triplicate.

### 4.4. Hyaluronidase Inhibition Assay

The hyaluronidase inhibitory activity of the extracts was assessed using the Hyaluronidase Inhibitor Detection Assay Kit purchased from Sigma-Aldrich (St. Louis, MO, USA), which employs a two-step turbidimetric reaction to measure the amount of hyaluronic acid hydrolyzed by the enzyme, where the decrease in turbidity is proportional to the enzymatic activity of the sample. The assay was conducted according to the manufacturer’s instructions, with slight modifications, using 96-well plates [70]. Briefly, a reaction mixture containing 10 μL of *C. odorifera* extract (HA-E, EA-E, B-E, and W-E, separately) and 20 μL of the enzyme (at a concentration of 100 U/mL in incubation buffer) was mixed in each well. After a 15 min incubation at 37 °C, a 20 μL substrate solution (0.1% sodium hyaluronate) was added to each well. The plates were incubated at 37 °C for an additional 20 min. Then, non-hydrolyzed hyaluronidase was precipitated by adding 250 μL of 2.5% cetyltrimethylammonium bromide (CTAB). The plates were incubated at 25 °C for 10 min. The intensity of complex formation was measured by absorbance at 630 nm in a spectrophotometer (METASH, UV-1500B). All samples were tested in quadruplicate. The percentage of hyaluronidase inhibition was calculated using Equation (2), where AS is the absorbance of the solution with the sample extract, AC is the absorbance of the solution without inhibitor, and AT is the absorbance of the enzyme.

Inhibition = (AS − AC)/(AT − AC) × 100(2)

### 4.5. Analysis of EA-E by High-Performance Liquid Chromatography Coupled to High Resolution Electrospray Ionization Mass Spectrometry (HPLC-HR-ESI-MS)

Because the highest anti-inflammatory activity was shown by the ethyl acetate extract (EA-E), it was analyzed by high-performance liquid chromatography (HPLC) coupled to a high-resolution electrospray ionization mass spectrometry (HR-ESI-MS), which was conducted using a Shimadzu HPLC system (Kyoto, Japan), equipped with a Shimadzu Diode Array Detector (DAD) (Kyoto, Japan). A sample concentration of 10 mg/μL of EA-E was separated using a Luna Phenomenex C18 column (4.6 × 250 mm, with a particle size of 5 μm and a pore size of 120 Å) (Phenomenex, Torrance, CA, USA). The mobile phase consisted of an 85:15 water: acetonitrile mixture, with a flow rate of 1.5 mL/min. Detection was performed at 237 nm. Mass spectrometry detection was performed in positive-ion mode using a Bruker micrOTOF-QII (Billerica, MA, USA) coupled to an Apollo ion source. The dry temperature was set at 200 °C, with a capillary voltage of 4.5 kV. The mass-to-charge ratios (*m*/*z*) were measured in both scan mode (*m*/*z* 100–1200 Da) and production scan mode (*m*/*z* 50–1200 Da) [71,72].

### 4.6. Isolation and Identification of the Main Compounds in EA-E

**Figure 4:**
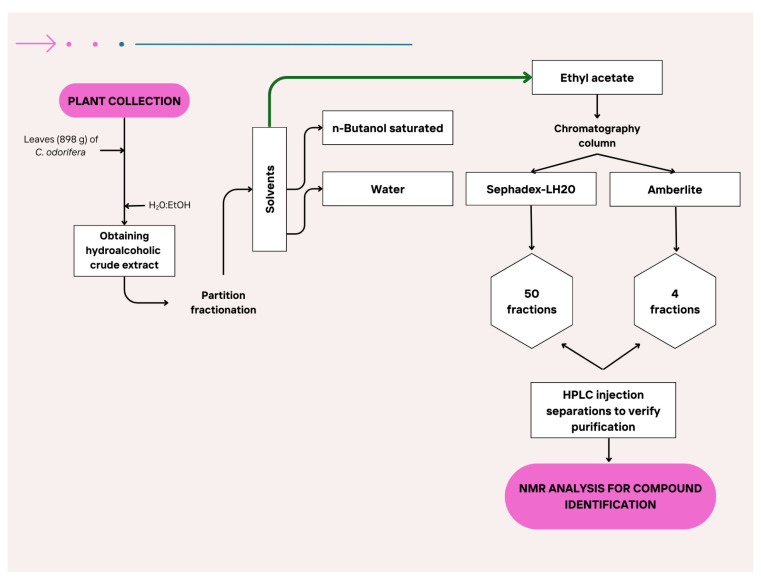
Isolation flow of compounds identified in the ethyl acetate fraction.

EA-E separation was performed by column chromatography. One gram of ethyl acetate extract was dissolved in methanol and placed at the top of a glass column (30 cm long × 5 cm diameter) packed with 100 mg of Amberlite (Sigma-Aldrich, St. Louis, MO, USA), which had been previously washed with an acidic water solution at pH 2. These fractions were lyophilized and dissolved in MeOH:H_2_O (1:1, *v*/*v*) for preparative HPLC-DAD analysis. Samples were filtered through a 0.22 µm PVDF membrane (Millipore) before injection. A UHPLC Thermo Ultimate 3000 system (Waltham, MA, USA) with a column XBridge^®^ C18 (5 µm, 250 × 10 mm) (Waters Corporation, Milford, MA, USA) was used to isolate pure compounds. The analysis was performed using a linear solvent gradient consisting of 1% formic acid (A) and acetonitrile (B) according the following gradient: 0–5 min, 15% B; 5–15 min, 30% B; 15–20 min, 50% B; 20–25 min, 100% B at a flow rate of 1 mL/min. Then, each fraction was analyzed by one-dimensional ^1^H and ^13^C NMR in a Varian (Palo Alto, CA, USA) (500 MHz) (Figure 4). Between 1 and 40 mg of compound fraction was dissolved in approximately 0.6 mL of DMSO-d_6_. These samples were filtered through a 45 µm porous diameter filter and then introduced into NMR tubes measuring 5 mm in diameter and 16.5/18 cm in length [73].

### 4.7. Statistical Analysis

Data were presented as mean ± standard deviation (s.d.). One-way ANOVA was used to test for overall differences. The significant ANOVA was followed by Duncan multiple comparisons to assess pairwise differences between treatment groups. A *p*-value of less than 0.05 was considered statistically significant.

## 5. Conclusions

This study demonstrated the significant anti-inflammatory potential of *Colliguaja odorifera*, an endemic Chilean plant traditionally used in folk medicine. The ethyl acetate extract inhibited key inflammatory enzymes, including LOX, hyaluronidase, and sPLA_2_. Phytochemical analysis revealed the presence of bioactive compounds, including shikimic acid, gallic acid, methyl gallate, ethyl gallate, and galloyl-luteolin, which may contribute to the observed pharmacological activity. According to Loncaric et al. [21], an IC_50_ of 11.75 µg/mL for LOX could be considered a strong-to-moderate response. However, these results need to be validated using cytotoxicity or cell-based bioassays. These findings support the traditional use of *C. odorifera* and suggest its potential as a natural source for developing safer alternatives to conventional anti-inflammatory drugs. Further studies are required to explore their mechanisms of action in detail. However, our results suggest that *C. odorifera* could inhibit the oxygenation in C-15 of unsaturated fatty acid and/or inhibit the hydrolysis of unsaturated fatty acids in the sn-2 position, such as oleic acid (C18:1) and linoleic acid (C18:2).

## Figures and Tables

**Table 1:** LOX inhibitory activity of *C. odorifera* extracts and isolated phenolic compounds.

Sample		LOX Inhibition (IC_50_ µg/mL)	Statistical Differences ^1^
Leaf extracts	HA-E	64.65 ± 12.92	a
	EA-E	11.75 ± 1.86	c
	B-E	42.50 ± 10.61	b
	W-E	NI	-
Pure compounds	Shikimic acid	0.97 ± 0.04	d
	Gallic Acid	0.37 ± 0.15	d
	Methyl gallate	60.70 ± 15.00	a
	Ethyl gallate	65.15 ± 12.45	a
Positive controls	Naproxen	0.56 ± 0.02	d
	Quercetin	0.48 ± 0.03	d

LOX: lipoxygenase; HA-E: hydroalcoholic extract; EA-E: ethyl acetate extract; B-E: Butanol extract; W-E: water extract. ^1^ Different letters indicate significant differences at *p* < 0.05. NI: No inhibition.

**Table 2:** Anti-inflammatory activity of *C. odorifera* leaves and isolated phenolic compounds.

Sample	sPLA_2_ Inhibition (IC_50_ µg/mL)	HA Inhibition (IC_50_ µg/mL)
EA-E	31.09 ± 4.59 a ^1^	6.60 ± 0.64 a ^1^
Gallic acid	11.16 ± 4.41 b	1.76 ± 1.31 b
Shikimic acid	NI	NI
Quercetin (positive control)	23.48 ± 3.85 a	1.80 ± 0.17 b

EA-E: ethyl acetate extract; NI: No inhibition. ^1^ Different letters indicate significant differences at *p* < 0.05.

**Table 3:** Mass spectrum and NMR data of compounds purified from the EA-E of *C. odorifera* leaves.

Peak	Name	RT (min)	[mg/G d.m.] ^1^	UV λmax	Molecular Weight (g/mol)	*m*/*z* Data	^1^H NMR (ppm)	^13^C NMR (ppm)
1	Shikimic acid	3.2	0.3	210	174	197 [M + Na]^+^ 157 [M-OH]^+^	δ 6.58 (s; H2) 4.21 (s; H3) 3.82 (m; H5) 3.57 (m; H4) 2.40 (d; H6a) 1.99 (d; H6b)	δ 168.07 (C1), 139.00 (C2), 128.36 (C3), 70.36 (C4), 66.87 (C5), 65.57 (C6), 29.94 (C7)
2	Gallic acid	5.0	2.7	272	170	193 [M + Na]^+^ 171 [M + H]^+^ 153 [M-OH]^+^	δH 6.88 (d; H2, H6)	δ 168.38 (C1),145.82 (C2), 138.65 (C3), 121.12 (C4), 109.67 (C5)
3	Methyl gallate	6.8	0.23	272	184	185 [M + H]^+^	δH 6.97 (s; H2, H6), 3.17 (s; H8)	δC: 120.40 (C1), 109.19 (C2, C6), 146.02 (C3, C5), 138.74 (C4), 168.21 (C7), 49.21 (C8)
4	Ethyl gallate	7.4	0.7	273	198	199 [M + H]^+^ 221 [M + Na]^+^	δH: 9.44 (H3, H5), 6.95 (s; H2, H6), 4.16 (s; H8), 1.21 (t; H9)	δC: 120.49 (C1), 109.33 (C2, C6), 145.59 (C3, C5), 139.01 (C4), 166.89 (C7), 61.06 (C8), 14.86 (C9)
5	Galloyl-luteolin	13.0	-	-	438	309 [luteolin + Na]^+^ 153 [gallic acid-OH]^+^	-	-

^1^ milligram per gram of dry matter.

**Table 4:** Lipoxygenase inhibition by extracts of plants of the family Euphorbiacea.

Species	Plan Part Extracted	Extracting Solvent	IC_50_ µg/mL	Control	IC_50_ µg/mL	Ref.
*Jatropha gossypifolia* L.	Leaf	Ethyl acetate	58.5	Baicalein	22.4	[23]
*Euphorbia neriifolia*	Stems	Hydro-alcoholic	73.1	-	-	[24]
*Euphorbia clavarioides* Boiss	Whole plant	Water	50.5	NDGA ^1^	0.45	[25]
*Croton zambesicus* Müll. Arg.	Bark	MeOH	93.7			[26]
*Mallotus oppositifolius* Müll. Arg.	Leaf	MeOH	39.2			
*Neoboutonia glabrescens* Prain	Leaf	MeOH	66.3		0.48	
*Drypetes gossweleri*	Bark	MeOH/CH_2_Cl_2_	46.7			

^1^ Nordihydroguaiaretic acid.

**Table 5:** Lipoxygenase inhibition by extracts of plants of species belonging to different families.

Species	Plan Part Extracted	Extracting Solvent	IC_50_ µg/mL	Control	IC_50_ µg/mL	Ref.
*Eryngium planum* L. (Apiaceae)	Leaf	MEOH	31.3	Ibuprofen	69.7	[27]
*Clerodendrum laevifolium* (Lamiaceae)	Leaf	Ethanol	14.1	Indomethacin	7.9	[28]
*Garcinia hombroniana* (Clusiaceae)	Leaf	Ethyl acetate	0.13	-	-	[29]
*Garcinia laterifolia* (Clusiaceae)	Leaf	Ethyl acetate	0.79	-	-	[30]
*Garcinia kydia* (Clusiaceae)	Leaf	Ethyl acetate	0.21	-	-	[31]
*Cyclea barbata* Miers (Menispermaceae	Leaf	Ethyl acetate	0.27	Baicalein	0.15	[32]

## Data Availability

Data are contained within the article.

## Abbreviations

The following abbreviations are used in this manuscript:

LOX	Lipoxygenase
sPLA_2_	Secretory phospholipase
HA	Hyaluronidase
EA-E	Ethyl acetate extract
B-E	Butanol extract
W-E	Water extract
